# Pre-Activation of Toll-Like Receptor 2 Enhances CD8^+^ T-Cell Responses and Accelerates Hepatitis B Virus Clearance in the Mouse Models

**DOI:** 10.3389/fimmu.2018.01495

**Published:** 2018-06-29

**Authors:** Yong Lin, Xuan Huang, Jun Wu, Jia Liu, Mingfa Chen, Zhiyong Ma, Ejuan Zhang, Yan Liu, Shunmei Huang, Qian Li, Xiaoyong Zhang, Jinlin Hou, Dongliang Yang, Mengji Lu, Yang Xu

**Affiliations:** ^1^Department of Microbiology, School of Basic Medicine, Tongji Medical College, Huazhong University of Science and Technology, Wuhan, China; ^2^Institute of Virology, University Hospital of Essen, Essen, Germany; ^3^Department of Infectious Diseases, Union Hospital, Tongji Medical College, Huazhong University of Science and Technology, Wuhan, China; ^4^State Key Laboratory of Organ Failure Research, Guangdong Provincial Key Laboratory of Viral Hepatitis Research, Department of Infectious Diseases, Nanfang Hospital, Southern Medical University, Guangzhou, China; ^5^Mucosal Immunity Research Group, State Key Laboratory of Virology, Wuhan Institute of Virology, Chinese Academy of Sciences, Wuhan, China

**Keywords:** toll-like receptor 2, hepatitis B virus, mouse model, proinflammatory cytokines, T-cell immunity

## Abstract

Toll-like receptors (TLRs) play a crucial role in activation of innate immunity, which is essential for inducing effective adaptive immune responses. Our previous studies have shown that toll-like receptor 2 (TLR2) is required to induce effective virus-specific T-cell responses against hepatitis B virus (HBV) *in vivo*. However, the contribution of TLR2 activation to adaptive immunity and HBV clearance remains to be clarified. In this study, we explored the hydrodynamic injection (HI) mouse models for HBV infection and examined how the TLR2 agonist Pam3CSK (P3C) influences HBV control and modulates HBV-specific T-cell response if applied *in vivo*. We found that TLR2 activation by P3C injection leads to the rapid but transient production of serum proinflammatory factors interleukin-6 and tumor necrosis factor-α and activation of CD8^+^ T cells *in vivo*. Then, the anti-HBV effect and HBV-specific T-cell immunity were investigated by TLR2 activation in the mouse models for persistent or acute HBV infections using HBV plasmids pAAV-HBV1.2 and pSM2, respectively. Both P3C application at early stage and pre-activation promoted HBV clearance, while only TLR2 pre-activation enhanced HBV-specific T-cell response in the liver. In the mouse model for acute HBV infection, P3C application had no significant effect on HBV clearance though P3C significantly enhanced the HBV-specific T-cell response. Collectively, TLR2 pre-activation enhances HBV-specific T-cell responses and accelerates HBV clearance in HI mouse models. Thus, the modulation of host immune status by TLR2 agonists may be explored for immunotherapeutic strategies to control HBV infection.

## Introduction

Hepatitis B virus (HBV) is a hepatotropic and non-cytopathic virus that causes more than one million deaths annually from liver cirrhosis and hepatocellular carcinoma (1, 2). The robust, polyclonal, and multispecific CD4^+^ and CD8^+^ T-cell response and neutralizing antibody responses contribute to the resolution of HBV infection. CD8^+^ T cells kill infected cells and secrete antiviral cytokines [mainly interferon gamma (IFN-γ)] that inhibit HBV replication non-cytopathically (3, 4). Neutralizing antibodies limit viral spread from residual productively infected hepatocytes (5).

Hepatitis B virus was at one time considered to be a “stealth virus” that induces negligible innate immune responses during the early phase of infection (6). Gene array analysis failed to detect an induction of host innate immunity during acute HBV infection in chimpanzees, as compared with HCV infection (7). A recent study in cell culture and a transplant mouse model also showed that HBV did not induce interferon (IFN) responses in hepatocytes but cytokine production in macrophages (8). However, there is accumulating evidence that the innate branch of the host immune system plays an important role in the control of HBV infection (9–11). Pattern recognition receptors, including toll-like receptors (TLRs), NOD-like receptors, RIG-I-like receptors, and leucine-rich repeat-containing receptors (NLRs), are essential for sensing invading pathogens, initiating innate immune responses, limiting the spread of infection, and orchestrating the activation and development of the adaptive immune response (12).

Among the PPRs, toll-like receptor 2 (TLR2) is widely expressed by antigen-presenting cells, endothelial and epithelial cells, and lymphocyte subsets, including activated and memory T cells (13, 14). TLR2 is a receptor for microbial pathogen-associated molecular patterns (PAMPs) like lipoproteins (12). TLR2 recognizes microbial PAMPs in its homodimeric form or alternatively as a heterodimer with TLR1 or 6 forms and activates various intracellular signaling molecules and transcription factors. PAMPs can bind to TLR2 receptor, which leads to cellular activation of Myd88-dependent signaling pathways (15). TLR2 stimulation leads to activation of cellular pathways including MAPK-ERK, AKT, and NF-κB signaling and suppression of hepadnaviral replication in hepatocytes and hepatoma cells (16–18). A downstream adaptor protein TAK1, if activated, can reduce HNF4α expression through the action of JNK pathway and thereby inhibit HBV replication (19). As a bridge between innate and adaptive immunity, TLR2 also contributes to the induction of specific immune responses to HBV infection and HBV clearance. In the HBV mouse model based on hydrodynamic injection (HI), TLR2 deficiency resulted in higher viral loads and reduced functionality of HBV-specific CD8^+^ T cells (20). The downstream signaling pathways of TLR2, mediated by MyD88/TRIF and IRAK4, are important for T-cell functions and complete viral clearance (20).

As TLR2 is expressed in activated and memory CD4^+^ and CD8^+^ T cells, it may serve as a co-stimulatory molecule to enhance T-cell proliferation, survival, and effector functions (10); thus, TLR2 deficiency impairs T-cell functions and viral control. Consistently, chronic HBV infection in patients is associated with reduced TLR2 expression in peripheral blood cells (PBLs) and in the liver (21, 22). In the woodchuck model, TLR2 expression on PBLs was decreased with increasing loads of woodchuck hepatitis virus (WHV) and recovered by suppressing WHV replication by antiviral treatment (17).

As TLR activation plays an important role for HBV control, it is attempted to explore TLR ligands for immunomodulation and treatment of chronic HBV infection (10, 23). Wu et al. applied poly(I:C), a TLR3 ligand, to mice with persistent HBV replication and found that poly(I:C) could stimulate immune responses in the mouse liver in an IFN-dependent manner and led to HBV clearance by recruiting HBV-specific T cells into the liver (24). Recently, Huang et al. showed that TLR9 ligand can induce recruitment of immune cells into the liver and formation of immune-related structures called intrahepatic myeloid cell aggregates for T-cell population expansion (iMATEs), which may support the local immune control of infections (25). Thus, we questioned whether and how TLR2 activation may promote specific immune responses to HBV proteins and contribute to HBV control. In the present study, we examined the immune activation by application of a potent TLR2 ligand and a synthetic lipopeptide-Pam3CSK4-in mice and how the immune activation promotes HBV control in the mouse models for chronic and acute HBV infections.

## Materials and Methods

### Mice

Male C57BL/6 mice aged 6–8 weeks were purchased from Beijing HFK Bioscience Co. Ltd (China) and were maintained under specific pathogen-free conditions in the Experimental Animal Center of Tongji Medical College, Huazhong University of Science and Technology. Animals were treated according to the Guidelines of the National Institutes of Health for Animal Care and Use.

### Plasmid and Peptides

Plasmid pAAV/HBV1.2 (kindly provided by Prof. Chen PJ, Graduate Institute of Clinical Medicine, College of Medicine, National Taiwan University, Taiwan) contains a terminally redundant 1.2× copy of genotype A HBV genome (26). Plasmid pSM2 (provided by Dr. Hans Will, Heinrich-Pette-Institute, Hamburg, Germany) is a replication-competent HBV clone harboring a head-to-tail tandem dimeric HBV genome (GenBank accession number: V01460). Both vectors were previously used by our group (24, 27).

The peptide of Kb-restricted HBV Cor93–100 epitope (MGLKFRQL) was synthesized and purchased from Chinese Peptide Company (Hangzhou, China). The synthetic lipopeptide Pam3CSK4 (P3C, TLR2/TLR1 ligand) was purchased from EMC Microcollections (Tübingen, Germany).

### Hydrodynamic Injection

Briefly, 10 µg of plasmid DNA (pAAV/HBV1.2 or pSM2) was hydrodynamically injected into the tail vein of C57BL/6 mice in a volume of phosphate-buffered saline (PBS) solution equivalent to 10% body weight within 5–8 s (24, 26, 27).

### Analysis of HBV Serological Markers and HBV DNA

Serum samples were collected from mice at the indicated time points after HI and diluted 1:10 with Diluent Universal (Roche Diagnostics, Switzerland). The levels of serum HBsAg, Hepatitis B e antigen (HBeAg), anti-HBc, anti-HBs, and HBV DNA were determined as described previously (24). Nucleocapsid HBV DNA was extracted from 60 mg mouse liver tissue according to the protocols published previously (28). The levels of HBV DNA were determined by SYBR Green Real-time PCR Master Mix (Toyobo, Japan). The following primers were used: forward primer, 5′-CTGCATCCTGCTGCTATG-3′ (nt 408–425) and reverse primer, 5′-CACTGAAC/AAATGGCAC-3′ (nt 685–701), based on the reference sequence with GenBank accession number AY220698.1.

### Detection of Cytokine Production by ELISA

Serum samples were collected from mice at the indicated time points after injection of P3C or PBS. The levels of serum interleukin-6 (IL-6) and tumor necrosis factor-α (TNF-α) were measured by specific Mouse cytokine ELISA kits (eBioscience, CA) according to the manufacturer’s instructions. Splenocytes were isolated from mice and cultured with αCD3/CD28 for 48 h. The IFN-γ levels in the supernatant were also measured by Mouse cytokine ELISA kits (eBioscience) in accordance with the manufacturer’s instructions.

### RNA Isolation and Real-Time RT-PCR

Total RNA was isolated from collected mouse liver tissue samples with Total RNA Kit (Omega) following the manufacturer’s protocol. One-step RT-PCR with real-time detection was carried out with the SYBR Green Real-time RT-PCR Master Mix (TaKaRa). The mRNA levels of IFN-β, IL-6, TNF-α, IL-10, and β-actin were detected by commercial QuantiTec Primer Assays (Qiagen) listed in Table S1.

### Lymphocyte Isolation From the Spleen and Liver

Murine splenocytes (SPLs) and intrahepatic lymphocytes (IHLs) were isolated as described previously (20). In brief, the mouse liver was perfused immediately with 10 ml PBS after sacrifice. After perfusion, the liver was homogenized and digested with enzyme solution containing 0.05% collagenase type II (Sigma-Aldrich), 0.002% DNAase I (Sigma-Aldrich), and 10% fetal bovine serum for 30 min. The pellet after digestion was resuspended in 40% Percoll and centrifuged at 1,000 *g* without break. After removing the debris and hepatocytes on the top layer, IHLs in the pellet were collected, washed, and subjected to further analysis. CD4^+^, CD8^+^, CD11b^+^, CD11c^+^, and F4/80^+^ cells in mouse spleen were isolated by appropriate microbeads purchased from Miltenyi Biotec GmbH.

### Cell Surface and Intracellular Cytokine Staining by Flow Cytometry

Surface and intracellular staining were performed as described previously (20). The antibodies used for surface staining included CD4, CD8, CD11c, CD25, CD43, CD100, and F4/80, were purchased from eBioscience. For intracellular cytokine staining, isolated SPLs and IHLs were seeded in 96-well plates and stimulated for 5 h at 37°C with 2 µg/ml selected CD8 T-cell epitope peptide in the presence of 2 µg/ml anti-CD28 antibody (BD Pharmingen) and 5 µg/ml of brefeldin A (eBioscience). The cells were primarily stained with anti-CD8 and 7-aminoactinomycin D (7AAD) (eBioscience), followed by intracellular cytokine staining using the Fixation & Permeabilization Kit (eBioscience) with the following antibodies: anti-IFN-γ (BD Pharmingen), anti-TNF-α (eBioscience), and anti-IL-2 (eBioscience).

For staining CD8^+^ T cells specific to the K^b^-restricted HBV Cor_93–100_ epitope, Recombinant Soluble Dimeric Mouse H-2K[b]: Ig Fusion Protein (DimerX I, BD Bioscience) was loaded with Cor_93–100_ overnight, and then used to stain mouse lymphocytes according to the technical instructions (27). The cells were first incubated with anti-CD16/CD32 rat anti-mouse antibody (BD Pharmingen) and then stained with anti-CD8 and 7AAD. After washing, the cells were incubated with dimer for 1 h, followed by staining with anti-IgG1 antibody (eBioscience) for 30 min at 4°C. Finally, stained cells were detected on FACS Calibur (BD Biosciences) and analyzed by using FlowJo software (Tree Star, OR).

### Statistical Analysis

Statistical analyses were performed using GraphPad Prism software version 5 (GraphPad Software Inc., CA, USA). Data were analyzed using nonparametric one-way ANOVA and Dunn’s multiple comparison test or Student’s *t*-test. *P*-values < 0.05 were considered significant.

## Results

### TLR2 Activation by P3C Application Leads to the Production of Pro-Inflammatory Factors *In Vivo* in the Presence of HBV Replication

**Figure 1:**
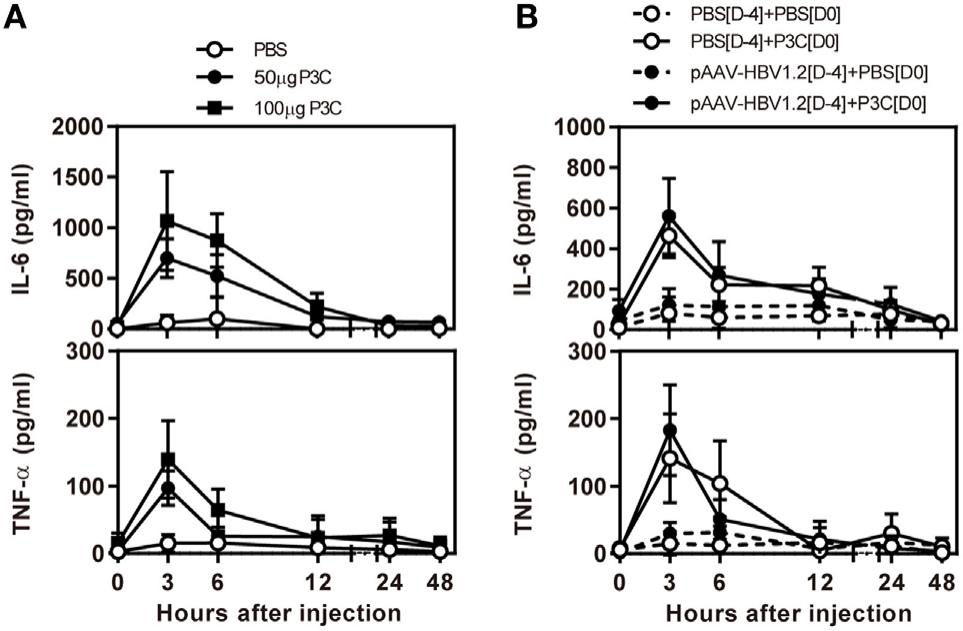
P3C treatment leads to the production of pro-inflammatory cytokines IL6 and tumor necrosis factor-α (TNF-α) *in vivo* with and without hepatitis B virus (HBV) replication. **(A)** C57BL/6 mice were treated once with 50 or 100 µg of Pam3CSK (P3C) or phosphate-buffered saline (PBS) subcutaneously administered at day 0. **(B)** C57BL/6 mice received hydrodynamic injection with plasmid pAAV-HBV1.2 4 days (D-4) before P3C treatment, followed by a single SC injection with 50 µg of P3C or PBS at day 0 (D0). The serum levels of the pro-inflammatory cytokines interleukin-6 and TNF-α were measured using specific ELISAs. Data were analyzed using an unpaired Student’s *t* test. Statistically significant differences between the groups are indicated as *P < 0.05 and **P < 0.01.

First, we examined the immune activation *in vivo* by application of TLR2 ligands P3C in C57BL/6 mice without and with HBV replication. C57BL/6 mice were treated with 50 and 100 µg of P3C by subcutaneous (SC) injection or PBS as control, respectively. The serum level of IL-6 and TNF-α was detected by specific ELISA at the indicated time points. The production of IL-6 and TNF-α was transient after P3C injection, with the levels peaking at 3 h, in a dose-dependent manner, and disappearing at 12 h after injection (Figure 1A). The plasmid pAAV-HBV1.2 was first hydrodynamically injected into C57BL/6 mouse to establish HBV replication (see below); then, 50 µg of P3C or PBS were subcutaneously injected at day 4 post-HI. The kinetic of the serum IL-6 and TNF-α production in mice with HBV replication had the same pattern like that in naive mice. Thus, the presence of HBV replication did not generally affect the production of pro-inflammatory cytokines IL6 and TNF-α stimulated by P3C (Figure 1B).

### Activation of TLR2 by P3C at Early Stage Inhibits HBV Replication in HBV-Persistent Replicating Mice

**Figure 2:**
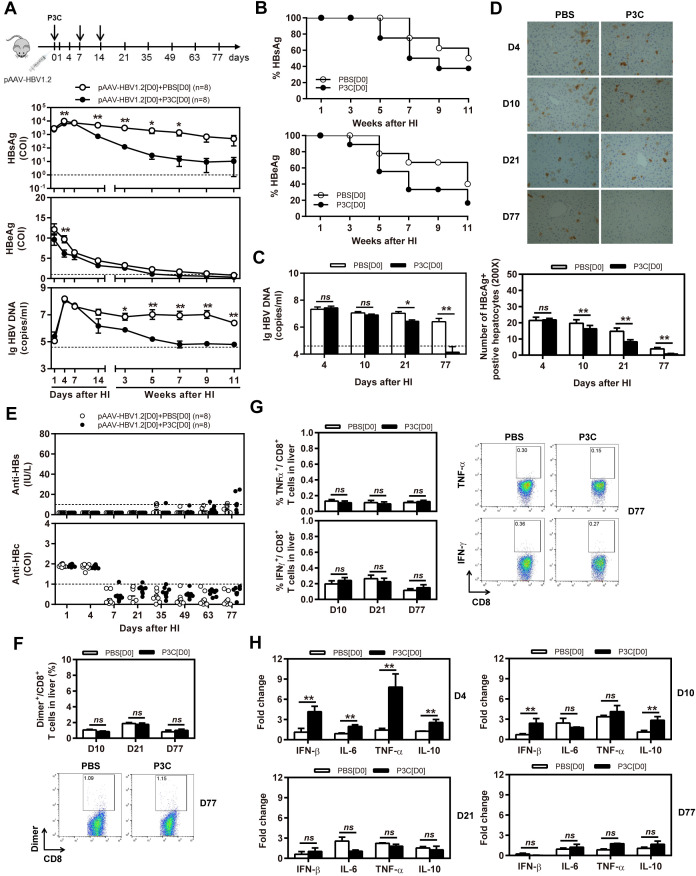
Early application of TLR2 ligand P3C with pAAV-HBV1.2 by HI inhibits HBV replication without promoting HBV-specific immune response in the mouse model for persistent HBV replication. C57BL/6 mice received hydrodynamic injection (HI) with plasmid pAAV-HBV1.2. The mice were treated three times with 50 µg of P3C or PBS administered by subcutaneous (SC) injection at day 0, 7, and 14 (therefore designated as group D0). **(A)** Serological markers of HBV infection HBsAg, HBeAg, and HBV DNA were assayed at the indicated time points by ECLIA (Roche). The cut-off value of the HBsAg and HBeAg assays was set at cut-off index (COI) of 1.0. The cut-off value of the HBV DNA real-time PCR was 4.0 × 10^4^ copies/ml. **(B)** Positivity for HBsAg or HBeAg was defined as ≥1. **(C)** HBV DNA levels in the liver were measured by quantitative real-time PCR. **(D)** Liver tissue sections were stained with anti-HBc antibodies (magnification, ×200). The number of HBcAg positive hepatocytes was counted. **(E)** The serum levels of anti-HBs and anti-HBc antibodies were detected at the indicated time points by ECLIA. The cut-off value of anti-HBs antibody assay was 10 IU/L. The cut-off value of anti-HBc antibody assay was 1.0 COI (<1.0 COI indicates a positive reaction). **(F, G)** Lymphocytes were isolated from the mouse liver at day 10, 21, and 77 after HI. **(F)** The specific CD8^+^ T cells against HBcAg Cor_93–100_ epitope were detected by Cor_93–100_ peptide-loaded dimer staining. **(G)** The functionality of HBV-specific CD8^+^ T cells was determined by intracellular cytokine staining after *ex vivo* stimulation with peptide Cor_93–100_ for 5 h. **(H)** Liver tissues were collected from the mouse liver at day 4, 10, 21, and 77 after HI. The mRNA expression levels of cytokines in the liver were determined by real-time RT-PCR. Beta-actin was used as an internal reference. Eight mice were analysed per group, and the experiments were repeated at least once. Data were analysed using an unpaired Student’s *t* test. Statistically significant differences between the groups are indicated as *P < 0.05 and **P < 0.01.

Next, we tested whether application of TLR2 ligand P3C would inhibit HBV replication in the HBV mouse model based on HI of pAAV-HBV1.2. In this model, HBV could replicate for a prolonged time *in vivo* (26). C57BL/6 mice were SC treated with 50 µg of P3C or PBS three times at days 0, 7, and 14 after HI of pAAV-HBV1.2 (D0 group). The kinetics of serum HBsAg, HBeAg, and HBV DNA indicated that the early P3C treatment at days 0, 7, and 14 inhibited HBV replication in mice (Figures 2A,B). While all mice treated with PBS remained positive for HBV infection markers, these markers gradually decreased in the mice of P3C treatment group and finally became negative in some individual animals. At day 77 after HI, serum HBV DNA and HBeAg were undetectable in P3C treated mice, while serum HBsAg was kept at low concentrations (<800 cut-off index, COI) in 37.5% of mice. Consistently, HBV DNA in mouse liver tissue samples was reduced by P3C treatment and was below the detection limit of the real-time PCR assay at day 77 (Figure 2C). The number of HBcAg- or HBsAg-positive hepatocytes in the mouse liver sections was detected by immunohistochemical staining and significantly decreased by P3C treatment as compared with PBS control (Figure 2D; Figure S1A in Supplementary Material). Anti-HBs antibody was positively tested in two P3C-treated mice at day 77, accompanied by the disappearance of serum HBsAg (Figure 2E). These results indicated that HBV could be cleared from some mice by P3C treatment in this model.

However, the frequencies and functionality of HBV-specific CD8^+^ T cells in the liver and spleen were not increased by P3C treatment at days 0, 7, and 14 (Figures 2F,G; Figures S1B,C in Supplementary Material), according to the results of staining with peptide Cor_93–100_-loaded dimers and intracellular cytokine staining. The cytokine production in the mouse liver was determined by real-time RT-PCR at day 4, 10, 21, and 77 after the first P3C application. The mRNA levels of IFN-β, proinflammatory cytokines IL-6 and TNF-α, and anti-inflammatory cytokine IL-10 were slightly higher than the control at day 4 (Figure 2H). It is not conclusive, which immune-related factor was responsible for HBV clearance.

**Figure 3:**
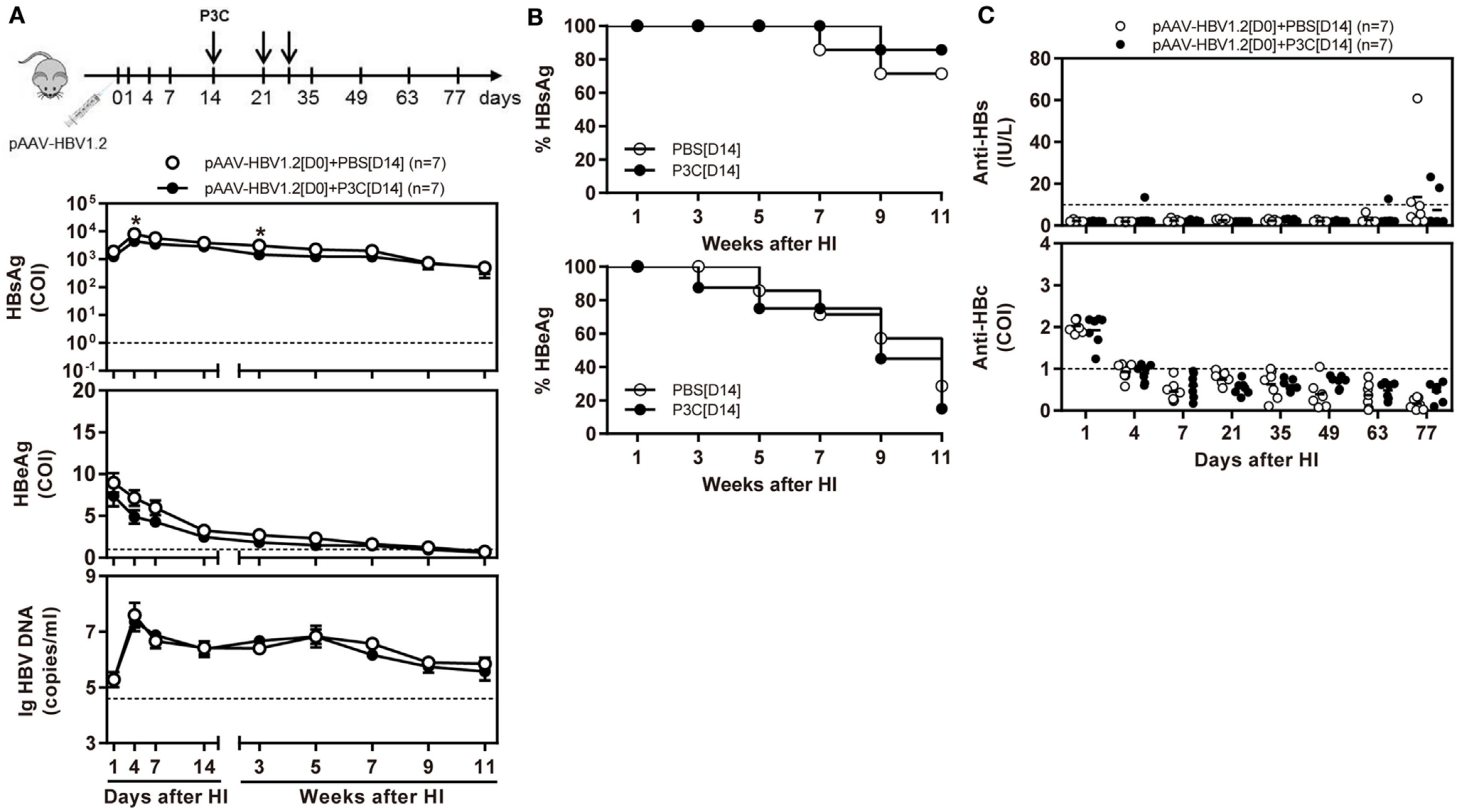
Application of toll-like receptor 2 ligand P3C at a late time point after hydrodynamic injection (HI) does not inhibit hepatitis B virus (HBV) replication in the model for persistent HBV replication. C57BL/6 mice received (HI) with plasmid pAAV-HBV1.2. These mice were then treated three times with 50 µg of P3C or phosphate-buffered saline administered by subcutaneous (SC) injection at day 14, 21, and 28 (D14). **(A)** Serological markers of HBV infection HBsAg, Hepatitis B e antigen (HBeAg), and HBV DNA were assayed at the indicated time points. **(B)** Positivity for HBsAg or HBeAg was defined as ≥1. **(C)** The serum levels of anti-HBs and anti-HBc antibodies were also detected at the indicated time points. Seven mice were analyzed per group, and the experiments were repeated at least once. Data were analyzed using an unpaired Student’s *t* test. Statistically significant differences between the groups are indicated as *P < 0.05 and **P < 0.01.

Besides, C57BL/6 mice were SC treated with 50 µg of P3C three times at days 14, 21, and 28 after HI (D14 group). The kinetics of serum HBsAg, HBeAg, and HBV DNA indicated that P3C treatment at the late time points had no effect on HBV replication in mice (Figures 3A,B). We found that 2 of 7 mice of the treated group spontaneously lost HBsAg and showed seroconversion to anti-HBs positive but with low anti-HBs antibody titers <20 IU/L. At the same time, one mouse of the control showed HBsAg seroconversion (Figure 3C). No statistical significance was found for HBsAg seroconversion and anti-HBs titers between the mouse groups.

Taken together, TLR2 activation at early time points after HBV HI inhibited HBV replication, likely by inducing the production of inflammatory cytokines, but not at late time points. Interestingly, the reduction and clearance of HBV replication was a relatively slow process, spanning over more than 10 weeks.

### Pre-Activation of TLR2 Accelerates HBV Clearance and Promotes HBV-Specific T-Cell Response in the Liver in the Mouse Model of Persistent HBV Replication

Our previous study with poly(I:C), a TLR3 ligand, demonstrated that direct application of TLR ligands into the liver may effectively recruit specific T cells and control HBV replication (24). Thus, we questioned whether TLR2 activation by intrahepatic application of P3C may enhance immune control of HBV infection.

**Figure 4:**
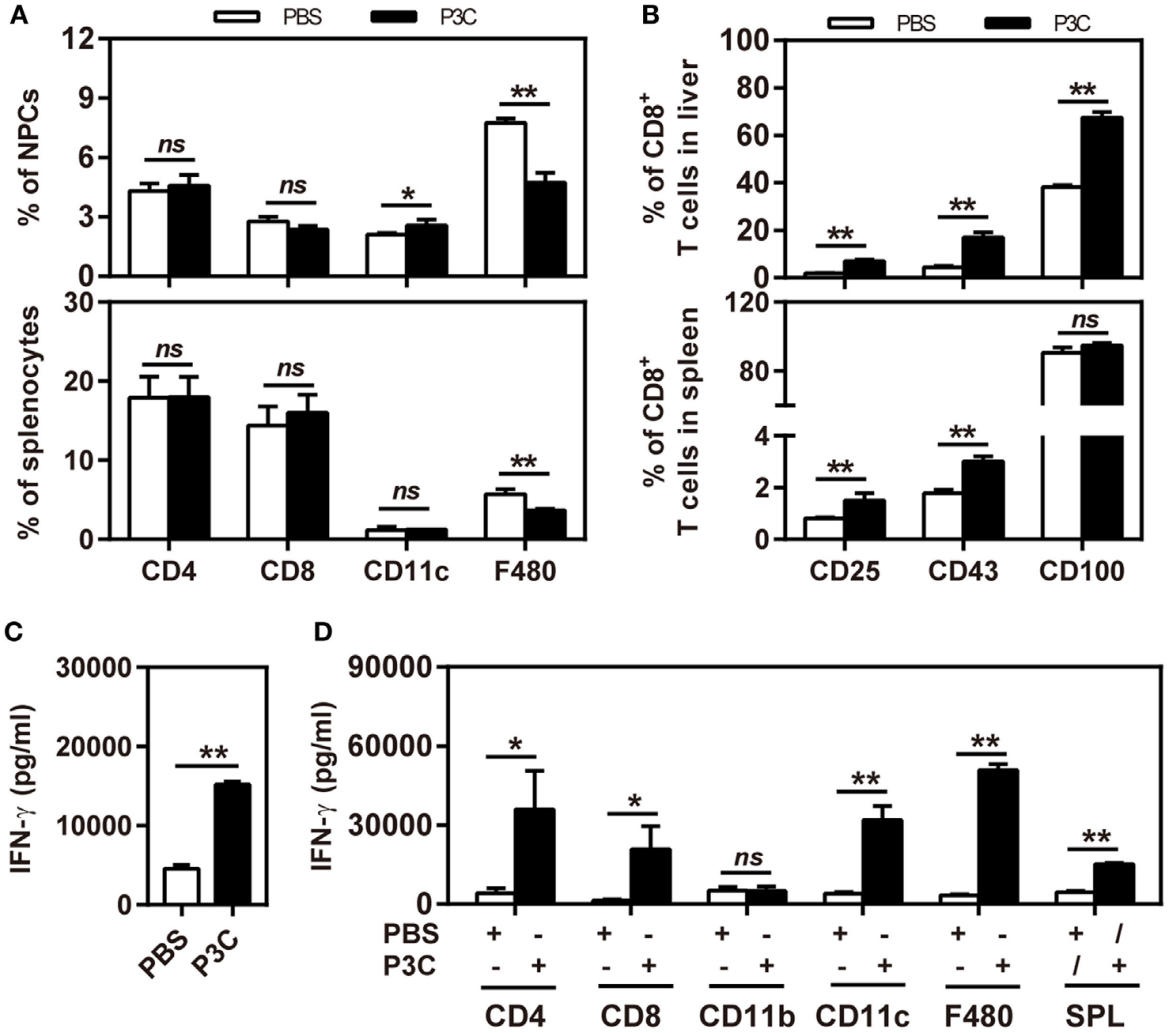
Application of P3C *in vivo* sustainably changes host immune status in the liver. **(A,B)** C57BL/6 mice were treated once with 20 µg of P3C or phosphate-buffered saline administered by intravenous (IV) injection. **(A)** Liver NPCs and splenocytes were separated at day 7 after IV injection of P3C. The composition of selected cell types was quantified by flow cytometry. **(B)** The surface markers CD25, CD43, and CD100 of CD8^+^ T cells from the liver and spleen were also detected by flow cytometry. **(C)** Murine splenocytes were isolated and stimulated with αCD3/CD28 for 48 h, and the supernatants were collected and subjected for detection of interferon gamma (IFN-γ). **(D)** The CD4^+^, CD8^+^, CD11b^+^, CD11c^+^, and F4/80^+^ cells in the spleen were positively isolated by appropriate microbeads (purchased from Miltenyi Biotec GmbH). CD4^+^, CD8^+^, CD11b^+^, CD11c^+^, and F4/80^+^ cells were exchanged between two groups and cultured with αCD3/CD28 for 48 h, and unfractionated splenocytes were used as a control. The supernatants were collected and subjected to the ELISA assay of IFN-γ. Data were analyzed using an unpaired Student’s *t* test. Statistically significant differences between the groups are indicated: * *P* < 0.05; **P < 0.01.

First, we treated C57BL/6 mice with 20 µg of P3C or PBS by intravenous (IV) injection. Liver NPCs and splenocytes were separated at day 7 after IV. The frequencies of CD4^+^, CD8^+^, CD11b^+^, CD11c^+^, and F4/80^+^ cells in the liver were analyzed by flow cytometry. We observed that the frequency of resident F4/80^+^ Kupffer cells in the liver was decreased. In contrast, the frequency of CD11c^+^ cells (DC) in the liver was slightly increased by P3C injection. However, the frequencies of CD4^+^ and CD8^+^ T cells in the liver and spleen were unchanged (Figure 4A). We further analyzed the expression of the activation markers of CD25, CD43, and CD100 on CD8^+^ T cells by flow cytometry. The results showed increased expression of these surface markers on CD8^+^ cells in the liver and spleen, indicating that P3C application led to a general immune stimulation and CD8^+^ T-cell activation (Figure 4B). We also examined whether TLR2 activation enhances the IFN-γ production of splenocytes activated by αCD3/CD28 stimulation. Indeed, higher levels of IFN-γ were produced by P3C-stimulated splenocytes than the control. Finally, we asked which cell types in splenocytes may contribute to IFN-γ production under TLR2 stimulation. To answer this question, we purified certain cell types CD4^+^, CD8^+^, CD11b^+^, CD11c^+^, and F4/80^+^ splenocytes from either P3C-treated or control mice, and separately cultured them with the rest of the splenocytes (CD4^−^, CD8^−^, CD11c^−^, CD11b^−^, or F4/80^−^) from either control or P3C mice. Then, IFN-γ production in the supernatant was determined after 48 h stimulation with αCD3/CD28. Interestingly, we found that higher levels of IFN-γ were produced by CD4^+^, CD8^+^, CD11c^+^, and F4/80^+^ cells after TLR2 activation Figures 4C,D.

**Figure 5:**
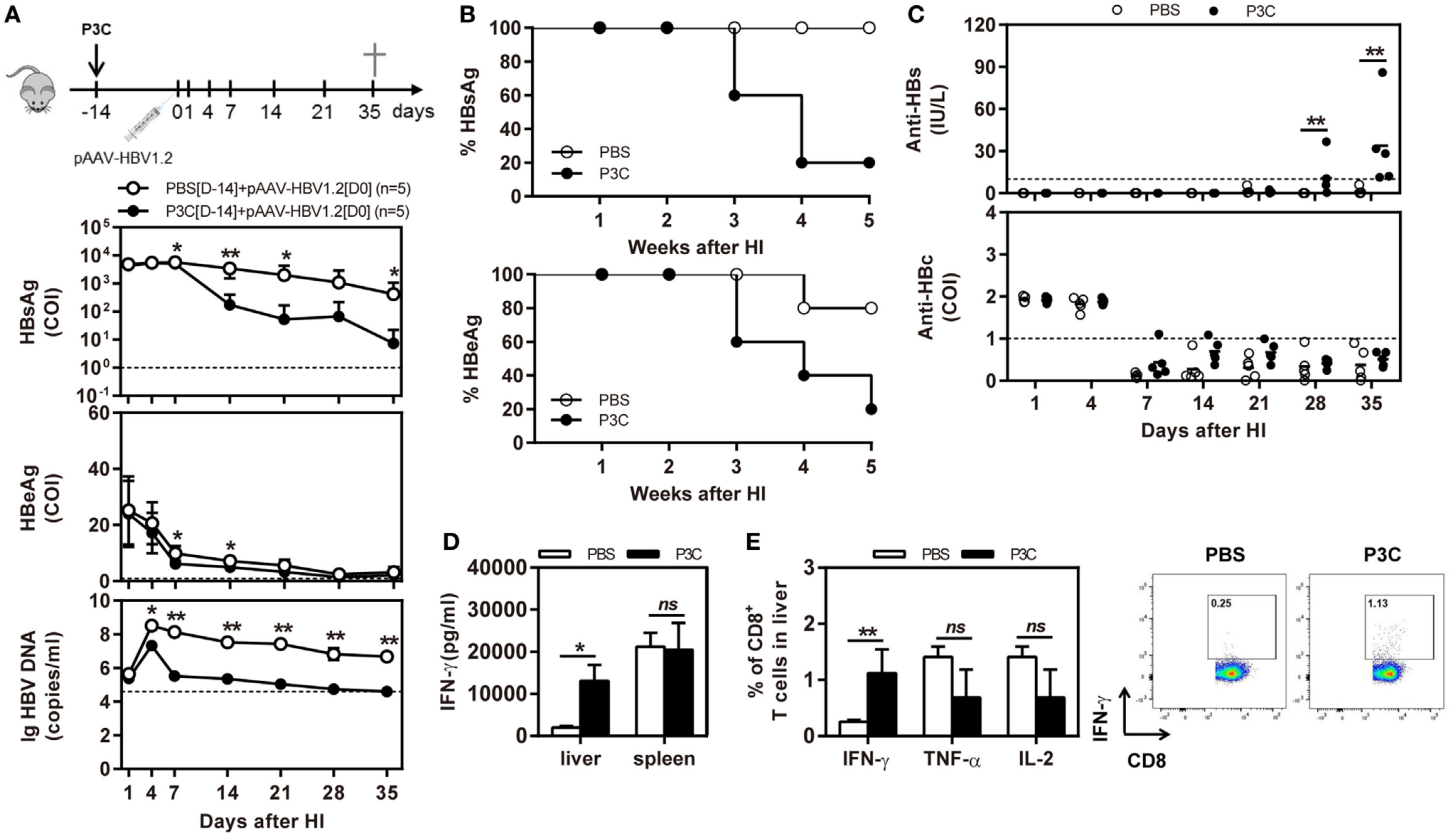
Pre-treatment with P3C accelerates hepatitis B virus (HBV) clearance in the mouse model for persistent HBV replication and promotes intrahepatic HBV-specific T-cell response. C57BL/6 mice were pretreated once with 20 µg of P3C or phosphate-buffered saline administered by intravenous (IV) injection at day 14 (D-14) before hydrodynamic injection (HI) with plasmid pAAV-HBV1.2. **(A)** Serological markers of HBV infection HBsAg, Hepatitis B e antigen (HBeAg), and HBV DNA were assayed at the indicated time points. **(B)** Positivity for HBsAg or HBeAg was defined as ≥1*. **(C)** The serum levels of anti-HBs and anti-HBc antibodies were detected at the indicated time points. **(D,E)** Lymphocytes were isolated from the mouse liver and spleen at day 35 after HI. **(D)** The lymphocytes were stimulated with peptide Cor_93–100_ for 48 h. Interferon gamma (IFN-γ) production was measured by ELISA. **(E)** The functionality of intrahepatic HBV-specific CD8^+^ T cells was determined by intracellular cytokine staining after *ex vivo* stimulation with peptide Cor_93–100_ for 5 h. The percentage of HBV-specific IFN-γ^+^ of CD8^+^ T cells was detected by flow cytometry. Five mice were analyzed per group, and the experiments were repeated at least once. Data were analyzed using an unpaired Student’s *t* test. Statistically significant differences between the groups are indicated as *P < 0.05 and **P < 0.01.

C57BL/6 mice were pretreated with 20 µg of P3C by IV injection at 14 days (D-14 group) before HI of pAAV-HBV1.2. HI with pAAV-HBV1.2 established prolonged HBV replication and gene expression in C57BL/6 mice, as described in the previous experiment (Figures 2A,B). By pretreatment with P3C, serum HBsAg, and HBeAg were cleared in 4 of 5 mice at day 35 after HI, along with decrease of serum HBV DNA below the detection limit (Figures 5A,B). Most importantly, anti-HBs antibody became positive in all P3C pre-treated mice at day 35 (Figure 5C). These results demonstrated that the P3C pre-treatment strongly promoted HBV clearance in mice that usually established persistent HBV replication. At day 35 after HI, lymphocytes were prepared from the liver and spleen and stimulated with HBcAg peptide *ex vivo*. The IHLs produced higher levels of IFN-γ (Figure 5D). Intracellular cytokine staining demonstrated that the frequencies of intrahepatic IFN-γ-producing CD8^+^ T cells were increased in response to *ex vivo* stimulation, while the fractions producing TNF-α and IL-2 did not change in a significant way (Figure 5E; Figure S2 in Supplementary Material). Collectively, the single injection of P3C at day 14 before HBV HI potently enhanced HBV-specific CD8^+^ T-cell response and led to rapid and efficient HBV clearance in the mouse model of persistent HBV replication.

### TLR2 Activation Enhances HBV-Specific T-Cell Response but Does Not Change the Kinetics of HBV Clearance in the Mouse Model of HBV Acute Infection

**Figure 6:**
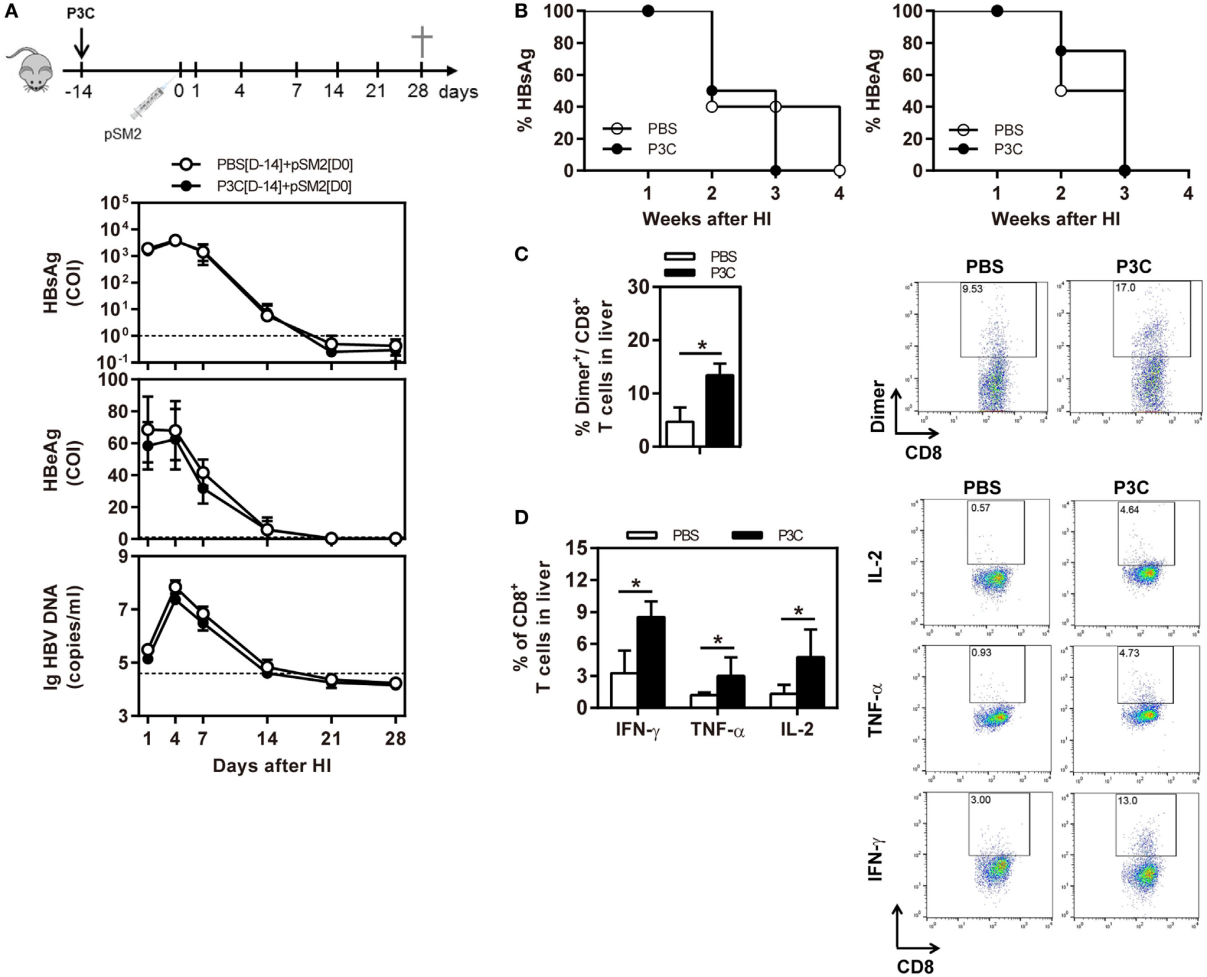
Pretreatment with P3C does not accelerate hepatitis B virus (HBV) clearance in the mouse model for acute HBV infection but promotes the HBV-specific T-cell response. C57BL/6 mice were pre-treated once with 20 µg of P3C or phosphate-buffered saline administered by intravenous (IV) injection at day 14 (D-14) before hydrodynamic injection (HI) with HBV plasmid pSM2. **(A)** Serological markers of HBV infection HBsAg, Hepatitis B e antigen (HBeAg), and HBV DNA were assayed at the indicated time points. **(B)** Positivity for HBsAg or HBeAg was defined as ≥1. **(C,D)** Lymphocytes were isolated from the mouse liver at day 28 after HI. **(C)** The specific CD8^+^ T cells against HBcAg Cor_93–100_ epitope were detected by Cor_93–100_ peptide-loaded dimer staining. **(D)** The functionality of intrahepatic HBV-specific CD8^+^ T cells was determined by intracellular cytokine staining after *ex vivo* stimulation with peptide Cor_93–100_ for 5 h. The HBV-specific IFN-γ^+^, TNF-α^+^, and IL-2^+^ CD8^+^ T cells was detected by flow cytometry. Four mice were analyzed per group, and the experiments were repeated at least once. Statistically significant differences between the groups are indicated as *P < 0.05 and **P < 0.01.

As pre-activation of TLR2 by P3C administration resulted in an accelerated HBV clearance in the mouse model for chronic HBV infection, we were interested to examine whether TLR2 pre-activation may also change the kinetics of HBV clearance in the mouse model for acute HBV infection. C57BL/6 mice were administered with 20 µg of P3C or PBS by IV injection at day 14 (D-14) before HI of plasmid pSM2. Again, the serum HBsAg, HBeAg, and HBV DNA disappeared in mice at day 21 after HI (Figures 6A,B), indicating that P3C treatment did not accelerate HBV clearance in mice. Similar to the previous experiments, P3C treatment significantly increased the frequencies of HBV-specific CD8^+^ T cells in the liver (Figure 6C). The HBV-specific CD8^+^ T cells from P3C-treated mice were also better in IFN-γ, TNF-α, and IL-2 production (Figure 6D). In conclusion, TLR2 activation by P3C promotes the HBV-specific T-cell response but does not show an effect on HBV clearance in the mouse model for acute HBV infection.

## Discussion

**Figure 7:**
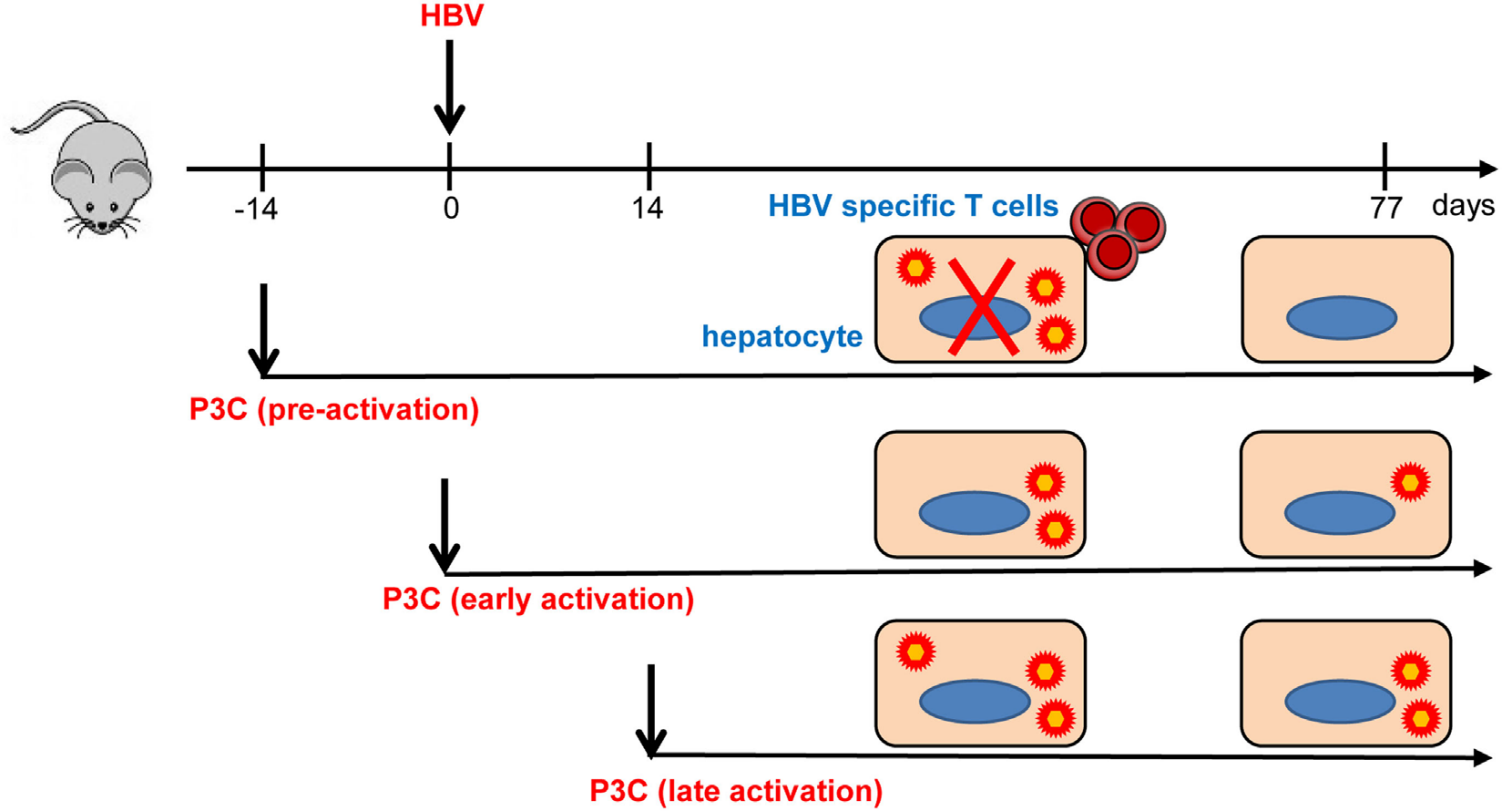
Toll-like receptor 2 (TLR2) activation promotes CD8^+^ T-cell responses and hepatitis B virus (HBV) clearance in mouse model. Pre-activation of TLR2 by P3C leads to enhanced HBV-specific CD8+ T cell responses and HBV clearance in the mouse model. P3C application at the early stage achieves HBV clearance in some mice in the model for persistent HBV replication. However, activation of TLR2 at a late time point does not inhibit HBV replication at all. TLR2 activation itself may only exert weak anti-HBV activity and could not provide help for HBV-specific T cell response if not prepared in advance.

In the present study, we investigated how TLR2 activation contributes to the control of HBV infection and immune response in the HBV HI mouse models. Our results have shown that pre-activation of TLR2 accelerates HBV clearance and promotes intrahepatic HBV-specific T-cell response in the chronic HBV mouse model (Figure 7). In the model for chronic HBV infection, early TLR2 activation led to viral suppression but without enhancing intrahepatic HBV-specific T-cell response, while no antiviral effect was observed if TLR activation occurred at a later time point.

Previously, we examined the role of TLR2 in HBV control and clearance in TLR2-deficient mouse model (20). Our data showed that lack of TLR2 expression led to elevated HBV replication and gene expression but did not delay HBV clearance. TLR2 deficiency resulted in impaired specific CD8^+^ T cell responses. However, HBV-specific CD8^+^ T cells were still recruited into the liver in a high level and produced IFN-γ that is required for HBV clearance (20). This work demonstrated the requirement of TLR2 for HBV control and clearance but did not indicate whether and how targeted TLR2 activation by ligands may promote host immune responses to HBV and HBV control. The present study gives answers to these remaining questions.

Our previous studies and those of other research groups showed that TLR2 activation in human hepatoma cells and primary woodchuck hepatocytes leads to reduction of HBV and WHV replication *in vitro* (16, 17). Activation of TLR2 signaling leads to transient production of pro-inflammatory cytokines that act directly as antiviral mediators, albeit with limited effect and may contribute to anti-HBV effect (17). However, this direct antiviral effect was rather weak as it did not lead the rapid HBV clearance in the chronic HBV mouse models (Figure 2) (29). Unlike TLR3 activation by poly(I:C), P3C application *in vivo* did not induce IFN production, rather only that of pro-inflammatory cytokines (24, 29, 30). Taken together, the direct antiviral effect of TLR2 activation is not the major factor for HBV control. Previously, we also found that the TLR2 ligand application did not change the course of WHV infection when woodchucks were acutely infected with WHV and treated early with TLR2 ligands, consistent with the results in the HBV mouse models (Figure S3 in Supplementary Material).

The activation of TLR signaling leads to transient production of pro-inflammatory cytokines, such as secreted IL-6 and TNF-α in serum (17, 24). Early TLR2 activation may trigger host innate immunity and production of pro-inflammatory cytokines (Figure 1). The pro-inflammatory cytokines produced in the host contribute to viral inhibition and reduce HBV replication in the liver (16, 17). Unfortunately, direct evidence is not yet available whether early TLR2 activation could enhance cellular immunity to clear HBV from the liver. We assume that other mechanisms like TNF-induced target cell killing may play a role to reduce HBV positive hepatocytes over time in this setting (31). Future studies will be needed to identify the exact antiviral mechanisms.

Although the antiviral effect of these cytokines is limited, they play a pivotal role for recruiting immune cells into the liver. The ultimate HBV clearance may mainly depend on HBV-specific CD8^+^ T cells in the liver, similar to the findings for TLR3 activation with poly(I:C) (25). We previously showed that TLR2 activation may also modulate the immune functions of intrahepatic liver sinusoidal endothelial cells (LSECs) (32). However, we further found that intrahepatic KCs retained the tolerizing activity after TLR2 activation and even increased the IL-10 production (33). Thus, the recruitment of immune cells into the liver, especially macrophages, may be essential to support T cells as effector cells against HBV (20). In this light, Huang described that iMATEs, which can attract activated CD8 T cells, are induced by intrahepatic application of TLR9 ligands (25).

Owing to the process of recruiting immune cells, a time lag is observed between TLR activation and HBV clearance in the liver (24). In the previous study, the application of poly(I:C) by HI led to transient production of type I IFN and induction of ISGs within hours. Although HBV replication decreased in the following days, the ultimate HBV clearance occurred only after CD8^+^ T cells were recruited in the dependence of CXCR3 into the liver. The peak of CD8^+^ cell infiltration into the liver was measured between days 20 and 30 after poly(I:C) application, which coincided with significant reduction of HBV loads (24). In the same line, TLR2 activation contributes to HBV control if the HI was performed 2 weeks later.

The fact that pretreatment of mice with TLR2 ligands led to enhanced specific T-cell responses and improved HBV control is consistent with the concept that the activation of innate immunity facilitates the initiation of adaptive immunity (34). Apparently, stimulation with TLR2 ligands gives a general activation of the host immune system and this enhanced activation status may be maintained for a while to respond to a possible invasion of pathogens. The immune activation is associated with the recruitment of various types of immune cells including macrophages, DCs, and T cells (24, 35). HBV HI into the liver with enriched immune cells triggered specific immune responses in a facilitated way. The recruitment of different immune cells into the liver occurs sequentially and requires a time period of more than 2 weeks for completion. Thus, TLR2 activation in the liver may accelerate HBV clearance through promoting the HBV-specific T-cell response but only if HBV infection is set after the completion of TLR2-mediated immune recruitment.

In the present study, we tested different ways to activate TLR2 by its ligands and measured the anti-HBV effects of these treatments. The routes and time points of the application of TLR2 ligands may determine the mode of immune activation in the hosts and the outcome of HBV control (24). Our results showed that the treatment of mice with P3C *via* SC application after the establishment of HBV replication was ineffective both for promoting adaptive immunity and viral suppression. The pre-activation of innate immunity by P3C injection significantly enhanced intrahepatic immunity and resulted in viral clearance in the mouse model of persistent HBV infection. These results presented here suggested the limitation and potential of TLR2-mediated immune activation to treat HBV infection and provide a guide for future studies.

## Ethics Statement

All animal experiments were conducted in a BSL-2 laboratory facility in accordance with the Guide for the Care and Use of. Laboratory Animals and were reviewed and approved by the Institutional Animal Care and Use Committee at Tongji Medical College, Huazhong University of Science and Technology, China (IACUC Number: 612).

## Author Contributions

ML and YX conceived and designed the study. YL, XH, JW, JL, MC, ZM, YL, SH, and QL performed the experiments and analyzed the data. YL, ML, and YX wrote the manuscript. EZ, XZ, JH, and DY provided materials and technical support and contributed to helpful discussions and review of the final manuscript. All authors read and approved the final manuscript.

## Conflict of Interest Statement

The authors declare that the research was conducted in the absence of any commercial or financial relationships that could be construed as a potential conflict of interest.

## Abbreviations

CHB, chronic hepatitis B; HBV, hepatitis B virus; HBsAg, Hepatitis B surface antigen; HBeAg, Hepatitis B e antigen; IFN-γ, interferon gamma; IL-6, interleukin-6; TLR2, toll-like receptor 2; PAMPs, pathogen-associated molecular patterns; PBMCs, peripheral blood mononuclear cells; PMA, phorbol-12-myristate-13-acetate; PRRs, pattern recognition receptors; TGF-β, transforming growth factor-beta; TNF-α, tumor necrosis factor-α.
