# Correction: Pre-activation of toll-like receptor 2 enhances CD8+ T-cell responses and accelerates hepatitis B virus clearance in the mouse models

**DOI:** 10.3389/fimmu.2025.1650574

**Published:** 2025-08-06

**Authors:** Yong Lin, Xuan Huang, Jun Wu, Jia Liu, Mingfa Chen, Zhiyong Ma, Ejuan Zhang, Yan Liu, Shunmei Huang, Qian Li, Xiaoyong Zhang, Jinlin Hou, Dongliang Yang, Mengji Lu, Yang Xu

**Affiliations:** ^1^ Department of Microbiology, School of Basic Medicine, Tongji Medical College, Huazhong University of Science and Technology, Wuhan, China; ^2^ Institute of Virology, University Hospital of Essen, Essen, Germany; ^3^ Department of Infectious Diseases, Union Hospital, Tongji Medical College, Huazhong University of Science and Technology, Wuhan, China; ^4^ State Key Laboratory of Organ Failure Research, Guangdong Provincial Key Laboratory of Viral Hepatitis Research, Department of Infectious Diseases, Nanfang Hospital, Southern Medical University, Guangzhou, China; ^5^ Mucosal Immunity Research Group, State Key Laboratory of Virology, Wuhan Institute of Virology, Chinese Academy of Sciences, Wuhan, China

**Keywords:** toll-like receptor 2, hepatitis B virus, mouse model, proinflammatory cytokines, T-cell immunity

In the published article, there was an error in **Figure 2** as published. Panel D of **Figure 2** appears in the published article by mistake. These errors have been corrected, and the updated panel D has been incorporated into the figure. The corrected **Figure 2** and its caption, appear below.

**Figure 2:**
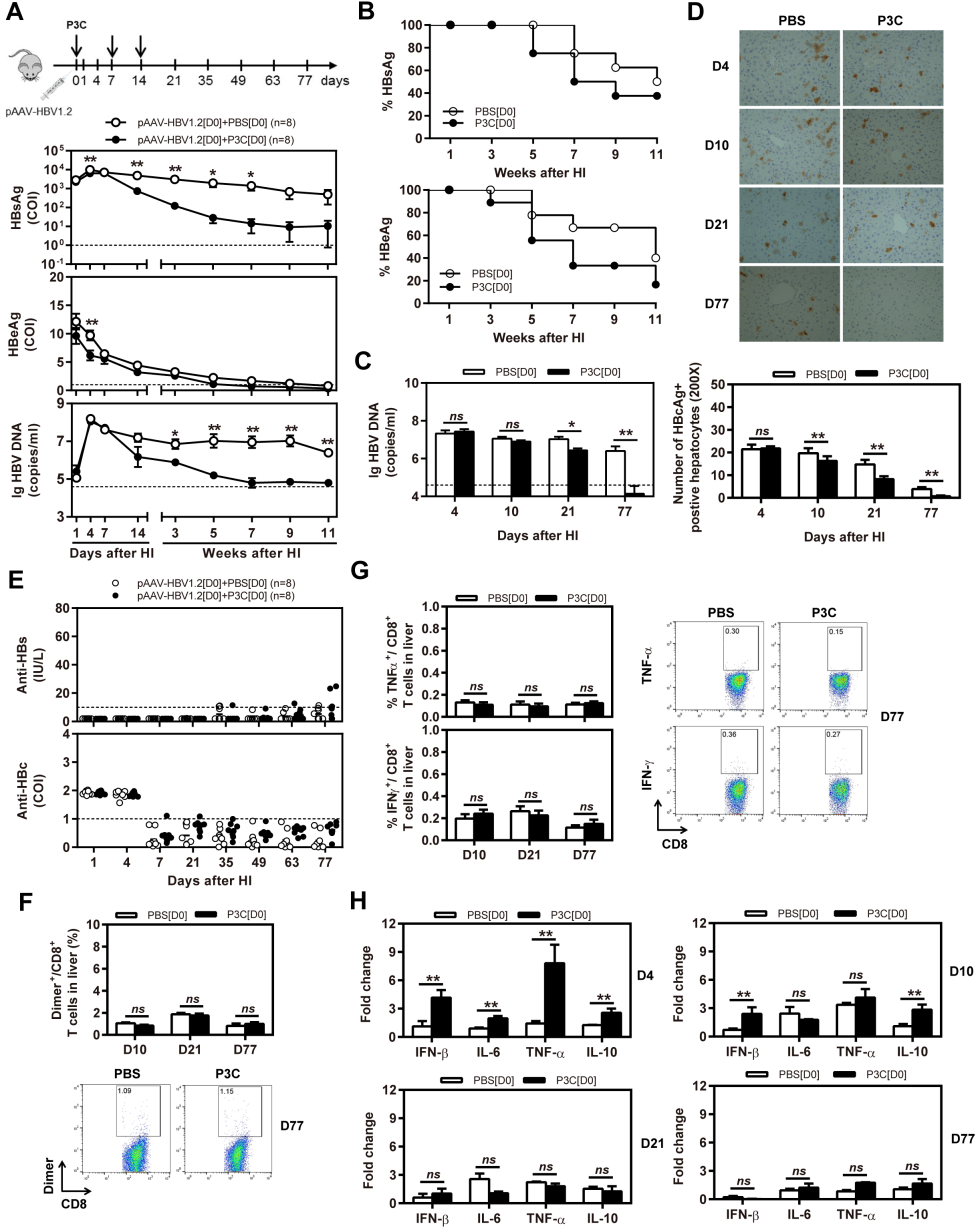
Early application of TLR2 ligand P3C with pAAV-HBV1.2 by HI inhibits HBV replication without promoting HBV-specific immune response in the mouse model for persistent HBV replication. C57BL/6 mice received hydrodynamic injection (HI) with plasmid pAAV-HBV1.2. The mice were treated three times with 50 μg of P3C or PBS administered by subcutaneous (SC) injection at day 0, 7, and 14 (therefore designated as group D0). **(A)** Serological markers of HBV infection HBsAg, HBeAg, and HBV DNA were assayed at the indicated time points by ECLIA (Roche). The cut-off value of the HBsAg and HBeAg assays was set at cut-off index (COI) of 1.0. The cut-off value of the HBV DNA real-time PCR was 4.0 × 10^4^ copies/ml. **(B)** Positivity for HBsAg or HBeAg was defined as ≥1. **(C)** HBV DNA levels in the liver were measured by quantitative real-time PCR. **(D)** Liver tissue sections were stained with anti-HBc antibodies (magnification, ×200). The number of HBcAg positive hepatocytes was counted. **(E)** The serum levels of anti-HBs and anti-HBc antibodies were detected at the indicated time points by ECLIA. The cut-off value of anti-HBs antibody assay was 10 IU/L. The cut-off value of anti-HBc antibody assay was 1.0 COI (<1.0 COI indicates a positive reaction). **(F, G)** Lymphocytes were isolated from the mouse liver at day 10, 21, and 77 after HI. **(F)** The specific CD8^+^ T cells against HBcAg Cor_93–100_ epitope were detected by Cor_93–100_ peptide-loaded dimer staining. **(G)** The functionality of HBV-specific CD8^+^ T cells was determined by intracellular cytokine staining after *ex vivo* stimulation with peptide Cor_93–100_ for 5 h. **(H)** Liver tissues were collected from the mouse liver at day 4, 10, 21, and 77 after HI. The mRNA expression levels of cytokines in the liver were determined by real-time RT-PCR. Beta-actin was used as an internal reference. Eight mice were analysed per group, and the experiments were repeated at least once. Data were analysed using an unpaired Student’s *t* test. Statistically significant differences between the groups are indicated as *P < 0.05 and **P < 0.01.

The authors apologize for this error and state that this does not change the scientific conclusions of the article in any way. The original article has been updated.

