# Supplementary material for: Pre-Activation of Toll-Like Receptor 2 Enhances CD8+ T-Cell Responses and Accelerates Hepatitis B Virus Clearance in the Mouse Models
Source: Front Immunol. 2018 Jun 29;9:1495. doi: 10.3389/fimmu.2018.01495 (PMC6033958; doi:10.3389/fimmu.2018.01495)
Supplement: Supplementary file 1 [file data_sheet_1.DOCX]

Supplementary Material

**Pre-activation of TLR2 enhances CD8^+^ T-cell responses and accelerates HBV clearance in the mouse models**

Yong Lin^1,2,3†^, Xuan Huang^1,4†^, Jun Wu^3^, Jia Liu^1,3^, Mingfa Chen^3^, Zhiyong Ma^1^, Ejuan Zhang^5^, Yan Liu^2^, Shunmei Huang^3^, Qian Li^1^, Xiaoyong Zhang^4^, Jinlin Hou^4^, Dongliang Yang^3^, Mengji Lu^1,2*^, Yang Xu^2*^

**^*^Corresponding authors:**

Prof. Dr. Mengji Lu: mengji.lu@uni-due.de

Dr. Yang Xu: orangexuyang@hotmail.com

1. **Supplementary Figures**

**
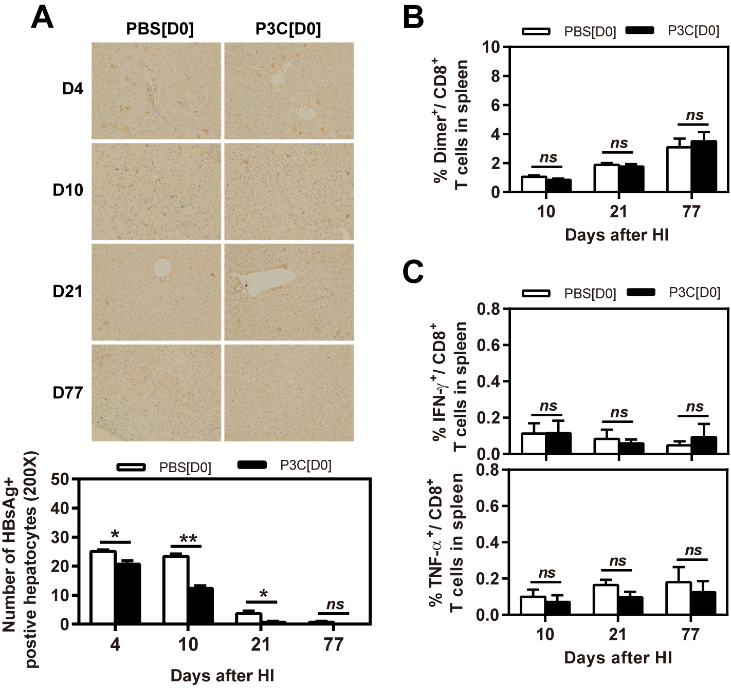
**

**Figure S1.** **Early application of TLR2 ligand P3C inhibits HBV replication in the HI mouse model for persistent HBV replication.** C57BL/6 mice received hydrodynamic injection (HI) with plasmid pAAV-HBV1.2. The mice were treated three times with 50 μg of P3C or PBS administered by subcutaneous injection at day 0, 7, and 14 (therefore designated as group D0). (A) Liver tissue sections were stained with anti-HBs antibodies (magnification, ×200). The number of HBsAg positive hepatocytes was counted. (B-C) Splenocytes were separated at day 77 after HI. (B) The specific CD8^+^ T cells against HBcAg Cor_93-100_ epitope were detected by staining with Cor_93-100_ peptide-loaded dimer and flow cytometry. (C) The functionality of HBV-specific CD8^+^ T cells was determined by intracellular cytokine staining after *ex vivo* stimulation with peptide Cor_93-100_ for 5 h. Data were analysed using an unpaired Student’s *t* test. Statistically significant differences between the groups are indicated as**P* < 0.05 and ***P* < 0.01; *ns*, not significant.

**
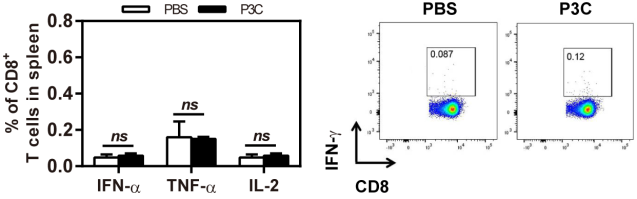
**

**Figure S2. Pre-treatment with P3C does not increase the frequencies of HBcAg-specific, IFN-γ- and TNF-α- producing CD8^+^ T cells in the spleen in the mouse model for persistent HBV replication.** Splenocytes were isolated at day 35 after hydrodynamic injection (HI) with plasmid pAAV-HBV1.2. The functionality of HBV-specific CD8^+^ T cells was determined by intracellular cytokine staining after *ex vivo* stimulation with peptide Cor_93-100_ for 5 h. Data were analysed using an unpaired Student’s *t* test. Statistically significant differences between the groups are indicated: *, *P* < 0.05; ** *P* < 0.01; *ns*, not significant.


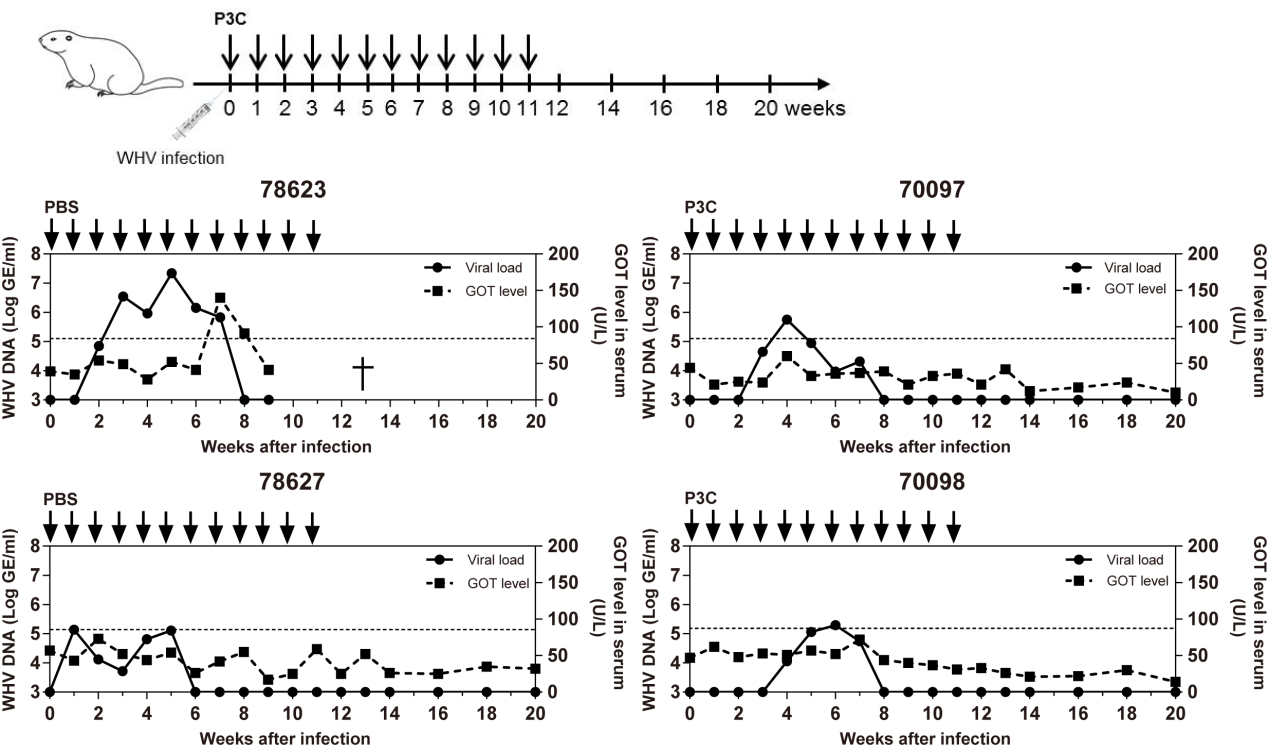


**Figure S3. P3C application in acutely WHV-infected woodchucks.** Four adult woodchucks were inoculated with an infectious dose with 1×10^7^ WHV genome equivalents. Then, the woodchucks were treated with 2 mg of P3C weekly for 12 doses through subcutaneous (SC) injection. PBS-treated woodchucks were used as control. The serum was collected from these woodchucks weekly for detection of WHV viral loads and glutamic-oxal(o)acetic transaminase (GOT).

1. **Supplementary Tables**

**Table S1**

**Primers for real time RT-PCR**

| **Name** | **Product Name** | **Company** | **Cat. No.** |
| --- | --- | --- | --- |
| beta-actin | Mm_Actb_2_SG QuantiTect Primer Assay | Qiagen | QT01136772 |
| IFN-β | Mm_Ifnb1_1_SG QuantiTect Primer Assay | Qiagen | QT00249662 |
| IL-6 | Mm_Il6_1_SG QuantiTect Primer Assay | Qiagen | QT00098875 |
| IL-10 | Mm_Il10_1_SG QuantiTect Primer Assay | Qiagen | QT00106169 |
| TNF-α | Mm_Tnf_1_SG QuantiTect Primer Assay | Qiagen | QT00104006 |

1. **Supplementary Materials and Methods**

**Woodchuck**

Woodchucks were purchased from North Eastern Wildlife (Ithaca, NY) and kept in the Central Animal Laboratory of University Hospital Essen. Relative animal experiments were conducted in accordance with the German Law for the Care and Use of Laboratory Animals, and the protocols were reviewed and approved by the District Government of Düsseldorf, Germany. Two naive and two acutely WHV-infected woodchucks were used in this study.

**Quantification of WHV DNA and GOT levels in the serum**

Serum WHV DNA and glutamic oxaloacetic transaminase (GOT) levels were quantified as described previously (1).

**Supplemental References**

1. Liu J, Zhang E, Ma Z, Wu W, Kosinska A, Zhang X, et al. (2014). Enhancing virus-specific immunity in vivo by combining therapeutic vaccination and PD-L1 blockade in chronic hepadnaviral infection. PLoS Pathog. 10(1):e1003856. doi:10.1371/journal.ppat.1003856
